# Comparative Global Gene Expression Profiles of Wild-Type *Yersinia pestis* CO92 and Its Braun Lipoprotein Mutant at Flea and Human Body Temperatures

**DOI:** 10.1155/2010/342168

**Published:** 2010-05-19

**Authors:** Cristi L. Galindo, Jian Sha, Scott T. Moen, Stacy L. Agar, Michelle L. Kirtley, Sheri M. Foltz, Lauren J. McIver, E. V. Kozlova, Harold R. Garner, Ashok K. Chopra

**Affiliations:** ^1^Department of Microbiology and Immunology, The University of Texas Medical Branch, Galveston, TX 77555-1070, USA; ^2^Virginia Bioinformatics Institute, Virginia Polytechnic Institute and State University, Blacksburg, VA 02461-0477, USA

## Abstract

Braun/murein lipoprotein (Lpp) is involved in inflammatory responses and septic shock. We previously characterized a Δ*lpp* mutant of *Yersinia pestis* CO92 and found that this mutant was defective in surviving in macrophages and was attenuated in a mouse inhalation model of plague when compared to the highly virulent wild-type (WT) bacterium. We performed global transcriptional profiling of WT *Y. pestis* and its Δ*lpp* mutant using microarrays. The organisms were cultured at 26 and 37 degrees Celsius to simulate the flea vector and mammalian host environments, respectively. Our data revealed vastly different effects of *lpp* mutation on the transcriptomes of *Y. pestis* grown at 37 versus 26°C. While the absence of Lpp resulted mainly in the downregulation of metabolic genes at 26°C, the *Y. pestis* Δ*lpp* mutant cultured at 37°C exhibited profound alterations in stress response and virulence genes, compared to WT bacteria. We investigated one of the stress-related genes (*htrA*) downregulated in the Δ*lpp* mutant relative to WT *Y. pestis*. Indeed, complementation of the Δ*lpp* mutant with the *htrA* gene restored intracellular survival of the *Y. pestis* Δ*lpp* mutant. Our results support a role for Lpp in *Y. pestis* adaptation to the host environment, possibly via transcriptional activation of *htrA*.

## 1. Introduction

*Yersinia pestis *is the causative agent of plague, and its current relevance as a potential bioweapon is garnered because of its high virulence and the development of multiantibiotic resistant strains by several governments prior to the 1972 Biological and Toxic Weapons Convention ban [1]. This gram-negative bacterium is naturally transmitted via a flea vector and prefers rodents as a reservoir. Plague can manifest itself in three different stages of disease progression: bubonic, septicemic, and pneumonic forms. Bubonic plague is the classic form where a flea vector bite leads to fever, headache, and the prototypical “buboes” or swollen lymph nodes in humans. Septicemic plague may result from a flea bite or inspiration; however, the disease progression leads quickly to high mortality with emesis, hemorrhagic rash, and high fever as its signs. Finally, pneumonic plague is spread person-to-person and is marked by fever, coughs, dyspnea, and hemoptysis. The aerosol is short-lived, as sunlight and desiccation destroy the bacterium. However, the dogma that *Y. pestis* is not hardy in the environment has been questioned as of late because it has been shown to remain viable on some fomites for over 72 hours [2] and can remain viable in the soil for approximately 40 weeks [3]. Even more notable than bacterial persistence is its virulence. 

A variety of virulence factor-encoding genes are found both on the chromosome and in plasmids. The pPCP1 plasmid contains the plasminogen-activating protease (Pla) which has been shown to interfere with the complement activation cascade and blood coagulation as well as decrease the extracellular matrix around the foci facilitating bacterial dissemination to peripheral organs [4]. Surface-bound Pla has also been shown to bind DEC-205 on phagocytic cells, which enhances bacterial uptake and consequently increases initial dissemination at the foci [5]. The plasmid pMT1 contains the murine toxin, which is integral for bacterial survival in the flea vector [6] and the F1 capsular protein, which protects the bacteria from phagocytosis [7] and possibly masks surface antigens from immune detection [8].

Another series of virulence factor-encoding genes, contained on the pCD1 plasmid, are the *Yersinia* outer membrane proteins (Yops) and a type three secretion system (T3SS) that translocates Yops. YopB, YopD, and the low calcium response antigen V (LcrV) have been shown to facilitate the translocation of the other Yops across the secretion apparatus, while some Yops act as effectors in the host cytoplasm [9]. For example, YopH is a protein tyrosine phosphatase that impedes the kinase-signaling cascades integral to the immune system's arbitration of infection [10]. YopT is a cysteine protease that cleaves the RhoA GTPase and consequently leads to the disruption of the actin cytoskeleton [11]. YopO is a Ser/Thr kinase that is activated by G-actin and also disrupts actin formation [12]. Similarly, YopE restricts signaling cascades [13] and acts with YopT and YopO to prevent internalization and subsequent MHC processing by phagocytic cells. YopJ also affects immune signaling cascades and has been shown to block the inflammasome [14] via acetylation of Ser/Thr residues, which blocks phosphorylation by MAPK kinases [15]. YopM has been shown to decrease the number of NK cells during infection [16]. Consequently, a therapeutic or vaccine therapy that can attenuate bacteremia prior to the translocation of high concentrations of these proteins into the host is desirable. Prior vaccine candidates, including the only licensed and now defunct Greer vaccine, have had limited success using whole bacteria. New specific markers on the immunopeptidome need to be investigated as candidates for a new generation of vaccines.

The Braun lipoprotein (Lpp) anchors the outer membrane to the peptidoglycan layer and is prevalent in many gram-negative enteric pathogens. Structurally, it acts as a spacer between the inner and outer membrane, keeping the periplasmic space open and helping to maintain outer membrane integrity [17]. Found on rough and smooth lipopolysaccharide (LPS) [18], we have recently examined the *Y. pestis* CO92 Lpp mutant in both bubonic and pneumonic murine plague models [19]. We have shown a statistical increase in animal survival utilizing this mutant, along with a decrease in general pathology of the tissues. Next, we examined the effect of this mutation on the host by analyzing the transcriptomes of mouse spleen, liver, and lungs during infection, using Affymetrix GeneChips. Overall, many immunological changes were seen in comparing the Lpp mutant with that of the WT bacterium [20]. Of note, many interferon (IFN)-*γ*-related genes were specifically down-regulated in the *lpp* mutant-infected mice. The Lpp mutant showed a general global decrease in transcriptional response by the host [20]. In conjunction with the effect bacteria have on the host transcriptome, the host also induces changes in the bacteria. 

In this study, we discerned if the mutation in the *lpp* gene would affect the bacterial transcriptome. Many conditions such as temperature, iron, and calcium have been shown to initiate large changes in *Yersinia* transcriptional regulation. Temperature and growth phase have recently been shown to heavily influence the production of outer member proteins [21], and nutrient exhaustion extensively regulates the transportation machinery [22]. Other studies have examined the effect of iron depletion [23], temperature extremes [24], and herbal remedies on the *Y. pestis* transcriptome [25]. We utilized bacterial microarrays to assess the host's ability to respond to Lpp, as it has been shown to initiate a toll-like receptor (TLR)-2 response and apoptosis in host cells [26]. Consequently, the effect of deletion of the *lpp* gene on the bacterial transcriptome will provide important information on how Lpp might modulate bacterial virulence at both flea and human body temperatures.

## 2. Materials and Methods

### 2.1. Bacteria Strains

WT *Y. pestis *CO92 was obtained from the Centers for Disease Control and Prevention (CDC, Atlanta, GA) and maintained in our restricted access biosafety level (BSL)-2 laboratory. The creation and characterization of the strain deficient in the expression of the *lpp *gene were described in detail previously [19]. All *Yersinia *strains were grown in either Brain Heart Infusion broth (BHI, Difco, Voigt Global Distribution Inc, Lawrence, KS) or Heart Infusion Broth (HIB, Difco) at 26–28°C [19, 27, 28].

### 2.2. Harvesting Bacterial RNA

 Prior to RNA isolation, bacteria were grown in BHI broth overnight at 26°C. The overnight culture was diluted 1  :  20 in BHI broth and grown at 26°C for an additional 6 hours. In another set of experiments, after 2 hours of cultivation at 26°C, the temperature was shifted to 37°C and calcium was added for 4 hours to facilitate activation of the T3SS and production of Yops. Bacteria were harvested and RNA isolated using RiboPure (Ambion/Applied Biosystems, Austin, TX). The experiments were performed in triplicate, with each microarray representing a separate biological culture of *Y. pestis*. Microarrays for *Y. pesti*s are available to our laboratory through the NIAID's Pathogen Functional Genomics Resource Center at The Institute for Genomic Research (TIGR), Rockville, MD. RNA was processed and hybridized by the Molecular Genomics Core Facility at UTMB, as we previously described [29, 30].

### 2.3. Microarray Data Analysis

Lowess normalization and statistical analyses were performed using GeneSpring GX 10 software (Agilent Technologies, Santa Clara, CA) as previously described [29]. Altered genes were deemed as significant if the fold-change was at least 1.5 and the *P*-value (based on Student's *t* test with Benjamini and Hochberg correction) was less than  .05. Hierarchical clustering was performed on normalized and log-transformed hybridization signals using CLUSFAVOR 6.0 (Baylor College of Medicine, Houston, TX). Raw and processed data (a total of 6 arrays) were deposited in the Gene Expression Omnibus (GEO) online (www.ncbi.nlm.nih.gov/geo) database (Accession GSE19840).

### 2.4. Amplification of the htrA Gene and Its in trans Expression in the Δlpp Mutant

The *htrA* gene (1443 bp) was amplified from the chromosomal DNA of *Y. pestis* CO92 by using polymerase chain reaction (PCR) and primers that targeted 200 bp upstream (5′CGCGGATCCTAGTA T GCAAAAATTTGAATTGTCCG3′-forward) and 53 bp downstream (5′ACGCGTCGACTG CATCTATTGTGTCAATACCTTAC3′-reverse) of the target gene. The PCR product was purified using the QIAquick PCR purification kit (Qiagen, Valencia, CA) and digested using the restriction endonucleases *Bam*HI and *Sal*I (New England BioLabs, Ipswich, MA) to prepare the fragment for ligation into pBR322 vector (Fermentas, Glen Burnie, MD) at the appropriate restriction enzyme sites. The target sites for these enzymes are underlined in the forward (*Bam*HI) and reverse (*Sal*I) primers, respectively. The resulting recombinant plasmid (designated as pBR322*htrA*) retains ampicillin resistance but is sensitive to tetracycline (Tc), as *Bam*HI/*Sal*I enzyme digestion removed the Tc cassette from plasmid pBR322. 

By using a similar strategy, a derivative of pBR322 plasmid (designated as pBR322Tc^s^), in which its Tc cassette was also removed by the *Bam*HI/*Sal*I digestion, was constructed and was used as control. The control plasmid pBR322Tc^s^ as well as pBR322*htrA* were then electroporated into their corresponding *Y. pestis* strains (i.e., WT and Δ*lpp Y. pestis* CO92 strains), respectively, by using a Gene Pulser Xcell (BioRad, Hercules, CA) in 2 mm cuvettes [31, 32]. The presence of the transformed plasmids in the corresponding* Y. pestis* strains was verified by plasmid isolation and restriction enzyme digestion.

### 2.5. Intracellular Survival of Y. Pestis Strains

*Y. pestis *strains were grown overnight in 3 mL of HIB broth at 28°C then stored at 4°C until the day of infection. Cultures were serially diluted and plated on trypticase soy agar plates with 5% sheep blood (SBA) in order to quantitate colony forming units (cfus) per mL. RAW 264.7 murine macrophages at approximately 3 × 10^6^ cells/well (70% confluence) were infected with *Y. pestis *CO92 strains at a multiplicity of infection (MOI) of 1, as previously described [19, 27, 28]. Plates were incubated at 37°C and 5% CO_2_ for 45 minutes to facilitate infection of RAW cells with the bacteria. After 45 minutes, infection medium was removed from the wells, and Dulbecco's modified Eagle's medium supplemented with 200 *μ*g/mL gentamicin was added to the monolayers to kill any extracellular bacteria. After 1 hour, the gentamicin-supplemented medium was replaced with medium containing a lower concentration of gentamicin (10 *μ*g/mL) in all plates except the 0-hour control plates for the duration of the experiment.

Beginning with the 0-h control plate, cells were harvested every 4 hours by the following method. After assessing the cell viability using light microscopy, the medium from each well was removed and the monolayers carefully washed 2X with phosphate-buffered saline (PBS). The cells were lysed using 300 *μ*L of ice-cold sterile water and released from the well using a sterile cell scrapper. The macrophage suspensions were serially diluted and cultured on SBA plates, which were then incubated at 28°C for 48 hours and the number of bacteria in each well was quantitated.

## 3. Results

### 3.1. Microarray Analysis of WT Y. Pestis CO92, Compared to Its Δlpp Mutant, Reveals Vastly Different Gene Expression Profiles at Vector and Mammalian Host Temperatures

WT *Y. pestis* CO92 and its Δ*lpp *mutant were cultured at 26 and 37°C, which represent the ambient temperatures of the flea vector and its mammalian hosts, respectively. To better understand how deletion of the *lpp* gene might influence gene expression, we performed two color microarrays on the *Y. pestis* CO92 Δ*lpp *mutant, compared to the WT strain at each temperature. The WT and mutant strains were grown simultaneously under identical conditions, and the experiment was performed in triplicate, resulting in six dual-color arrays (3 arrays for bacteria grown at 26°C and 3 arrays at 26°C and then shifting the temperature to 37°C). Lowess normalization was performed on all three arrays for each temperature set using GeneSpring GX10 microarray analysis software, and hybridization signals below background were removed before further analysis. Student's *t* test with Benjamini and Hochberg correction was performed, and only those genes with a *P* value ≤.05 that were differentially expressed in the mutant bacteria by at least 1.5-fold for each replicate were considered as significantly altered. 

**Figure 1:**
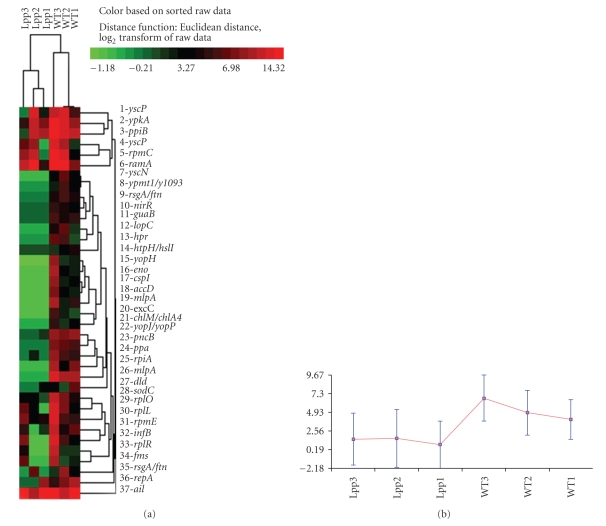
Hierarchical clustering of genes determined to be significantly altered in the Δ*lpp* mutant of *Y. pestis* CO92 cultured at 26°C relative to the WT bacteria. (a) Heat map showing clustering of genes differentially expressed between the *Y. pestis *Δ*lpp* mutant, compared to WT bacteria, is presented. Clustering was performed on normalized and log-transformed hybridization signals using CLUSFAVOR 6.0 (Baylor College of Medicine, Houston, TX). The three replicate samples representing the two experimental conditions (*Y. pestis* CO92 or its Δ*lpp* mutant) are labeled as WT and Lpp, respectively. Note that the two experimental conditions clustered apart from one another, and altered genes collectively exhibited a pronounced difference in signal intensity. The vertical dendrograms indicate relative similarity between samples (columns), while the horizontal dendrograms indicate clusters of genes (rows). Bright red indicates the highest normalized intensity value, bright green the lowest, and black median values. (b) Graphical representation of the cluster shown in panel (a). Normalized signal intensity values are shown on the ordinate, and experimental conditions are listed on the abscissa. The blue bars represent the range of normalized, log-transformed signal intensities for the entire group of genes while the red line indicates the median signal and thus the trend of gene expression differences. As shown, the average and median signal intensities for this group of genes is lower in the *Y. pestis *Δ*lpp* mutant, compared to the WT bacteria.

Based on these criteria, there were 51 genes that exhibited significant reduction in expression at 26°C in the Δ*lpp* mutant, compared to WT *Y. pestis* CO92 (Table 1). Hierarchical clustering produced a heat map that similarly demonstrated significant differences in expression for these genes and also successfully separated the three replicate experiments for the Δ*lpp* mutant and WT strains, as shown in Figure 1. As expected, the *lpp* gene itself (listed as *mlpA* in Table 1) was identified by the analysis as down-regulated 362-fold in the Δ*lpp* mutant, compared to WT *Y. pestis* CO92 (Table 1). The majority of genes that were down-regulated in the absence of *lpp* (at 26°C) were those involved in various metabolic processes (e.g., protein and nucleic acid synthesis and energy production). There were also nine virulence genes, mainly T3SS components, and effectors that were down-regulated in the Δ*lpp* mutant, compared to WT *Y. pestis*, when both were grown at 26°C (Table 1). Interestingly, there were no genes more highly expressed in the mutant grown at 26°C, compared to WT *Y. pestis *cultured under the same conditions. 

When the temperature was shifted from 26°C to 37°C and the experiment was repeated, there were only 39 genes that were significantly altered in expression in the Δ*lpp* mutant, compared to WT *Y. pestis* based on microarray analysis (Table 2). The gene encoding Lpp was significantly repressed (−1,138-fold) in the mutant, compared to WT *Y. pestis* CO92, as was found for the Δ*lpp* mutant grown at 26°C (Table 1). In addition to the *lpp* gene, there were three other genes that were similarly reduced in expression in the *Y. pestis* CO92 Δ*lpp* mutant when cultured at 26°C or first at 26°C and then at 37°C: two hypothetical genes encoding proteins and the gene encoding D-lactate dehydrogenase (data not shown). The remaining 35 gene expression alterations were specific to 37°C, suggesting that Lpp plays a role in bacterial gene expression that occurs during the transition between *Yersinia's* flea vector and the mammalian host environment.

**Figure 2:**
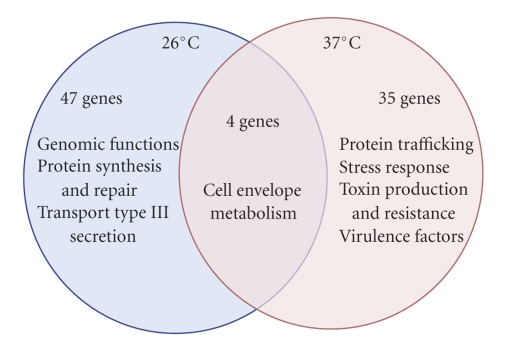
Venn diagram showing the overlap of major functions of genes identified as significantly altered in a Δ*lpp* mutant of *Y. pestis* CO92 and the WT strain. Functions were obtained from the CMR online database (http://cmr.jcvi.org) as well as from the literature. Numbers of genes significantly altered (at least 1.5-fold, Benjamini and Hochberg-corrected *P* value ≤.05) exclusively and commonly in the Δ*lpp* mutant of *Y*. *pestis* CO92 cultured at 26°C and at 37°C, compared to its respective WT control, are also shown.

Another notable difference between bacteria grown at 26 and 37°C was that the transcriptome of the Δ*lpp* mutant cultured at 37°C included four upregulated genes: two stress response genes, one gene that codes for a putative membrane protein and a gene whose product is involved in gamma-aminobutyrate metabolism (Table 2). Most interesting was the nearly complete lack of overlap in gene functions between bacteria grown at 26°C and those cultured at 37°C in the absence of the *lpp* gene. The only functions that the two gene sets had in common were cell envelope structure maintenance and metabolism (Figure 2). Only the Δ*lpp* mutant that was shifted from 26°C to 37°C exhibited alterations (mainly downregulation compared to WT *Y. pestis*) in genes important for protein secretion and trafficking (non-T3SS components), stress response, toxin production and resistance, and other notable virulence factors (Table 2 and Figure 2). For example, the iron-sulfur cluster assembly genes *iscR* and *nifS* were both down-regulated in the Δ*lpp* mutant compared to the WT bacterium (2-fold and 1.9-fold, resp., Table 2). Other down-regulated stress response genes included *htpN* (reduced 3.5-fold) *degQ* (reduced 1.9-fold) and *htrA* (reduced 2.9-fold, Table 2). We also noted down-regulation of *qacE* and *ymoA* genes (reduced 4.1- and 2.2-fold, resp., Table 2), which are important for bacterial virulence. 

### 3.2. Intracellular Survival of Y. pestis CO92 Δlpp Mutant Is Restored Following Complementation with the htrA Gene

RAW murine macrophages were infected with *Y. pestis *strains at an MOI of 1. At 0, 4, 8, and 12 hours post infection (p.i.) monolayers were lysed, harvested, and cultured on SBA plates to enumerate the number of bacteria in each well. Our previous studies have shown that lack of the *lpp *gene decreases the ability of this mutant bacterium to survive within the harsh environment of macrophages [19, 27]. As our microarray analysis data showed, there was a decrease in the *htrA* gene expression in the Δ*lpp *mutant after temperature shift as compared to WT-infected cells (Table 2). This gene (also referred to as *gsrA*) was previously shown to be important for intracellular survival of *Y. enterocolitica* in macrophages [33, 34]. Therefore, we hypothesized that complementation of the Δ*lpp* mutant with the *htrA *gene *in trans* would facilitate the restoration of intracellular survival of this mutant in macrophages.

**Figure 3:**
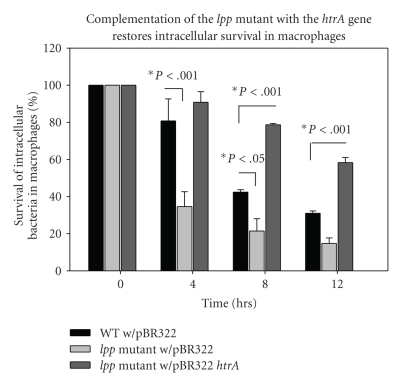
Complementation of the *lpp* mutant of *Y. pestis* CO92 with the *htrA* gene restores intracellular survival in macrophages. Intracellular survival of WT with pBR322, Δ*lpp* mutant with pBR322, and the Δ*lpp* with pBR322*htrA* was determined by infecting RAW 264.7 murine macrophages with an MOI of 1 for 45 minutes, followed by a 60-minute gentamicin wash, and plating the surviving intracellular bacteria at 4, 8, and 12 hours. The Δ*lpp* mutant with pBR322*htrA* has a significant increase in percent survival compared to the Δ*lpp* mutant with pBR322 alone, as determined by ANOVA and Holm-Sidak method.

At 4 hours p.i. (Figure 3), the number of *Y. pestis *Δ*lpp *mutant bacteria containing only the control plasmid pBR322Tc^s^ recovered from the macrophages significantly decreased as compared to those from macrophages infected with the WT bacteria harboring the control pBR322Tc^s^ plasmid. The number of *Y. pestis *Δ*lpp *mutant bacteria containing the pBR322/*htrA* plasmid that were recovered from macrophages, however, did not decrease after 4 hours of infection and, in fact, was about 10% higher than the number of WT bacteria containing the pBR322Tc^s^ plasmid recovered from macrophages. At 8 hours p.i., the recovery of *Y pestis *Δ*lpp *mutant bacteria was significantly lower than that of WT bacteria-infected macrophages. Additionally, the number of Δ*lpp *mutant bacteria containing the pBR322/*htrA* plasmid recovered from macrophages was significantly higher than that of WT bacteria-infected cells. This trend continues through 12 hours p.i., indicating a possible protective effect from the harsh environment of macrophages on *Y. pestis *Δ*lpp *mutant bacteria following complementation with the *htrA *gene.

## 4. Discussion

In the present study, we compared the global transcriptomes of a Δ*lpp* mutant of *Y*. *pestis* CO92 to the WT strain at 26°C and by shifting temperature from 26 to 37°C, we demonstrated that vastly different transcriptional responses to the *lpp* gene deletion occurred under the two different temperature conditions. At 26°C, which simulates the flea vector temperature conditions, mainly metabolic genes were altered in response to the *lpp* gene deletion, compared to WT *Y*. *pestis*. More interesting was the down-regulation of the T3SS components, as well as the *ail* (attachment-invasion locus) gene, which encodes a virulence-associated outer membrane protein that promotes invasion of epithelial cells [35], inhibits the antibody-mediated classical pathway of complement activation via binding the complement regulator C4b-binding protein [36], and is uniquely expressed in virulent strains of *Y. enterocolitica* [37]. 

In *Y. pestis*, the Ail protein was recently shown to mediate binding and delivery of Yop proteins to human epithelial cells and human monocytes [38] and was additionally demonstrated to be critical for *Y. pestis* infection of mice [38]. It is unclear why these potent virulence factor-encoding genes would be expressed at 26°C in the presence of the *lpp* gene since this temperature simulates the environment in the flea where type 3 secretion and mammalian immune evasion are not needed. It is possible that Lpp plays a role in directly or indirectly modulating the function of these genes; however, we did not observe any alteration in T3SS components or *ail* when the temperature was shifted to 37°C to mimic the temperature of the *Y. pestis* during infection of the mammalian host. It is possible that transcriptional regulation of T3SS components is related to differential modification of Lpp at these two temperatures, a phenomenon which has been demonstrated for LPS [39]. Our previous studies, in which we observed differential sensitivity of *Y. pestis* CO92 to polymyxin B when cultured in vitro versus in vivo [28], support this hypothesis, which we plan to further investigate in future. Polymyxin B appears to bind differentially to various forms of LPS produced by *Y. pestis* under in vitro versus in vivo growth conditions, thus showing differential susceptibility of bacteria to this antibiotic in these two environments.

While survival of *Y. pestis* in its flea vector is only peripherally related to pathogenicity in humans, it is nonetheless a critical part of the infective cycle, and some genes that are important for virulence might also play a role in survival outside the mammalian host. The protein encoded by *guaB* for instance, which was reduced in expression by 22-fold in *Y. pestis *Δ*lpp* mutant compared to the WT strain at 26°C, is a key enzyme in the purine salvage pathway that seems to play a dual role in *Borrelia burgdorferi*. The activity of GuaB was recently shown to be essential for *B. burgdorferi* infection of mice and was also demonstrated to provide a growth advantage to the bacteria in the tick [40]. The authors concluded that GuaB is critical for the survival of *B. burgdorferi* in the infection cycle and that there are likely differences in the requirements for purine salvage in the tick and mammalian environments. Our results support their conclusions and further suggest that Lpp plays a role in adaptation to the different metabolic needs of *Y*. *pestis* in the flea and mammalian environments. 

In contrast to what was observed for the Δ*lpp* mutant cultured at 26°C, *Y. pestis* that was grown in the absence of *lpp* at 37°C exhibited a significant perturbation in the transcription of multiple stress response and virulence genes (Table 2 and Figure 2). Also altered in expression were genes related to Type VI secretion and the regulation of protein translocation and trafficking (Table 2). Some of the genes altered in the absence of the *lpp* gene at 37°C play multiple roles related to cell survival and virulence. For example, the *iscR* gene, which was down-regulated in the *Y. pestis *Δ*lpp* mutant as compared to the WT bacterium (Table 2), encodes a transcriptional regulator that controls the expression of genes required for the biosynthesis of iron-sulfur clusters. In *E. coli*, iscR controls iron-dependent biofilm formation [41], and more generally the ISC system is important for survival during oxidative stress and in response to iron deprivation [42]. Likewise, the gene encoding NifS that was down-regulated in the Δ*lpp* mutant compared to WT *Y. pestis* (Table 2) is also a regulator of iron-sulfur cluster biosynthesis and is important for the survival of various bacteria in hostile environments [43, 44].

There were three genes involved in toxin production and/or antibiotic resistance that were down-regulated in the Δ*lpp* mutant at 37°C, as compared to WT *Y. pestis*. One of these genes encodes QacE (Table 2), which was shown to be associated with multiple resistances to antibiotics and antiseptics in clinical isolates of *Enterobacter cloacae*, *Citrobacter freundii*, *Pseudomonas aeruginosa*, and *Stenotrophomonas maltophilia* [45], as well as environmental and clinical isolates of *Vibrio parahaemolyticus* and *V. cholerae* [46]. Two genes related to spheroplast formation (pesticin and a gene similar to the *Bacillus subtilis* stage V sporulation protein R) were down-regulated in the Δ*lpp* mutant, compared to WT *Y. pestis *(Table 2). YmoA, also down-regulated in expression in the *Y. pestis *Δ*lpp* mutant compared to WT bacteria (Table 2), has been shown to negatively regulate the expression of *Y. enterocolitica* [47] and* pseudotuberculosis* [48] invasin, important for the initiation of infection. The importance of invasin in these two species of *Yersinia *is understandable as these are gastrointestinal pathogens. However, since *Y. pestis* directly enters the blood stream, the importance of this invasin gene in plague needs to be further explored. 

The vast majority of genes that were altered in expression in the *Y. pestis *Δ*lpp* mutant cultured at 37°C, compared to WT bacteria, were those related to survival during stress, including increases in temperature. For example, the gene encoding HtpN was down-regulated 3.5-fold in the *Y. pestis *Δ*lpp* mutant, relative to the WT strain (Table 2), and this protein is important for survival of *E. coli *at higher temperatures (up to 41.5°C) [49] and has additionally been proposed to play a role in* E. coli* biofilm formation [50]. The expression of the gene encoding DegQ was reduced by 1.9-fold in the Δ*lpp* mutant compared to WT *Y. pestis* (Table 2). This protein was originally identified in *E. coli* as an essential component for growth at elevated (30+ °C) temperatures [51], and mutations in the *degQ* gene were also shown to affect survival of *Salmonella enterica* serovar Typhimurium in the host [52]. Similarly, HtrA is a heat-shock inducible chaperone and protease, and the human homologue can additionally associate with microtubules and thereby inhibit cell migration [53]. In *Streptococcus pneumoniae*, HtrA regulates bacteriocin activity [54], and when mutated in *Mycobacterium tuberculosis*, it attenuates virulence in mice [55]. In this study, we demonstrated that HtrA can restore the ability of the *Y. pestis *Δ*lpp* mutant to survive in macrophages (Figure 3). This suggests that Lpp-mediated intracellular survival of bacteria in host immune cells is mediated through the activation of the *htrA* gene transcription. 

Interestingly, the *IcrQ/yscM* genes, which code for putative T3SS regulatory proteins, were down-regulated in the Δ*lpp* mutant of *Y. pestis* CO92 compared to its WT bacterium(5.3-fold).The potential function of these regulatory genes is to block transcription of the *yop* genes.However, our recent studies indicated that the T3SS was intact in the Δ*lpp* mutant and it translocated YopH and YopE effectors into HeLa cells similar to that of WT bacteria [19]. Further, HeLa cells infected with the WT and mutant bacteria exhibited similar T3SS-associated cytotoxicity [19]. Consequently, the role of these protein secretion and trafficking genes in the context of transcription of *yop* genes needs to further investigated.

## 5. Conclusions

 This study provided the first comprehensive assessment of the global effects of *lpp* gene mutation on *Y. pestis* CO92 gene expression, as well as a comparison of the *Y. pestis *Δ*lpp* mutant transcriptomes at 26°C versus 37°C, which simulate the flea vector and mammalian host environments, respectively. Our results support a role for Lpp in survival of *Y. pestis* in the harsh environment of the host and the switch in gene expression from mainly metabolic functions to stress response and virulence genes when the temperature of growth was shifted from 26 to 37°C. We additionally propose that inhibition of intracellular survival of *Y. pestis *Δ*lpp* mutant in macrophages is mediated via repression of *htrA* gene transcription, based on our ability to restore survivability of **Δ** *lpp* mutant of *Y. pestis* CO92 complemented with the *htrA* gene. This study underscores the importance of performing experiments such as these at the host temperature and comparing gene expression alterations under different culturing conditions. Finally, our data tend to suggest that the *lpp *gene may also have a regulatory role in addition to its role as a structural gene.

## Figures and Tables

**Table 1:** Transcripts down-regulated in a Δ*lpp* mutant of *Y. pestis* CO92 cultured at 26°C, compared to wild-type bacteria, based on microarray analyses.

Yer ID	Gene Name	Gene Symbol	Function	Δ*lpp*/WT
				FC

*Cell envelope*				

YPO2394	major outer membrane lipoprotein	*mlpA*	*lpp* gene—cell envelope	−361.9
YPO1125	peptidoglycan-associated lipoprotein Pal	*excC*	Maintenance of cell envelope integrity	−22.3
YPO0448	putative lipoprotein	—	Cell envelope	−26

*Genomic functions*				

y2424	putative transposase	—	Mobile and extrachromosomal element functions	−76.8
Y1119.1n	replication protein A	*repA*	DNA replication, recombination, and repair	−31.9
YPO1968	transposase for insertion sequence IS100	*ypmt1*/y1093	Mobile and extrachromosomal element functions	−22.3

*Metabolism*				

YPO2705	conserved hypothetical protein	—	Fermentation	−93.1
YPO2805	putative aldo/keto reductase	—	Central intermediary metabolism	−20.3
YPO3387	conserved hypothetical protein	—	Nitrogen fixation	−16.7
y1601	acetyl CoA carboxylase, carboxytransferase component, beta subunit	*accD*	Fatty acid and phospholipid metabolism	−48.3
YPO1161	molybdopterin [*mpt*] converting factor, subunit 1	*chlM/chlA4*	Biosynthesis and degradation of surface polysaccharides and lipopolysaccharides	−15.7
YPO1177	D-lactate dehydrogenase	*dld*	Fermentation	−6.4
YPO3376	enolase	*eno*	Glycolysis/gluconeogenesis	−127.7
y1362	IMP dehydrogenase	*guaB*	Purine salvage pathway	−22
YPO2993	PTS system, phosphocarrier protein	*hpr*	Degradation of proteins, peptides, and glycopeptides	−29.3
YPO2329	D-lactate dehydrogenase	*htpH/hslI*	Fermentation	−7
YPO2300	fumarate and nitrate reduction regulatory protein	*nirR*	Regulation of metabolic functions	−15.7
YPO1413	putative nicotinate phosphoribosyltransferase	*pncB*	Biosynthesis of cofactors, prosthetic groups, and carriers	−51.7
YPO3521	inorganic pyrophosphatase	*ppa*	Central intermediary metabolism	−44.1
YPO0915	ribose 5-phosphate isomerase A	*rpiA*	Pentose phosphate pathway	−124.7

*Protein synthesis and repair*				

YPO0242	polypeptide deformylase	*fms*	Protein modification and repair	−4.1
y0688	protein chain initiation factor IF-2	*infB*	Translation	−30.2
YPO3074	peptidyl-prolyl cis-trans isomerase B	*ppiB*	Protein folding and stabilization	−405.1
YPO0233	30S ribosomal protein S4	*ramA*	Protein synthesis	−3.2
YPO3748	50S ribosomal protein L7/L12	*rplL*	Protein synthesis	−6.7
YPO0228	50S ribosomal protein L15	*rplO*	Protein synthesis	−11.4
YPO0225	50S ribosomal protein L18	*rplR*	Protein synthesis	−8.7
YPO0218	50S ribosomal protein L29	*rpmC*	Protein synthesis	−11.6
y0299	50S ribosomal subunit protein L31	*rpmE*	Protein synthesis	−15.1

*Transport*				

YPO2672	putative urea transporter	—	Transport and binding proteins	−33.3
YPO3156	ATP-dependent Clp protease ATP-binding subunit ClpX	*lopC*	Carbohydrate transport	−29.3
YPO1783	ferritin	*rsgA/ftn*	Iron transport	−12.4

*Type III Secretion System*				

YPO2905	attachment invasion locus protein	*ail*	Invasion of eukaryotic cells; Type III Secretion System	−3.3
YPCD1.67c	putative protein-tyrosine phosphatase Yop effector	*yopH*	Type III secretion effector protein	−100.7
YPCD1.71c	putative targeted effector protein	*yopJ/yopP*	Type III secretion effector protein	−68.2
				FC

YPCD1.72c	putative targeted effector protein kinase	*ypkA*	Type III secretion effector protein	−21.2
YPCD1.40	putative Yops secretion ATP synthase	*yscN*	Type III secretion system component	−30.7
YPCD1.42	putative type III secretion protein	*yscP*	Type III secretion system component	−66.9

*Other functions*				

y0223	cold shock-like protein	*cspI*	Response to cold shock	−68.7
YPO1363	putative virulence factor	—	Pathogenesis	−34.8
y0815	superoxide dismutase precursor (Cu-Zn)	*sodC*	Resistance to reactive oxygen species	−18.2

*Unknown functions*				

various genes (y3398, YPO0130, 0198, 1087, 1560, 1996, 2153, 2854, and 3699)				*

Functions were obtained from the CMR online database (http://cmr.jcvi.org) and from the literature. FC = fold-change, which was calculated as the ratio between the hybridization signals for that gene in the *Y. pestis Δlpp* mutant and WT bacteria (*Δlpp*/WT). Expression differences were deemed as statistically significant if the fold-change was ≥1.5 and *P* value ≤.05. A negative sign (“−”) before the FC indicates down-regulation of the gene in the *Y. pestis Δlpp* mutant, relative to the WT strain. *FCs for genes with unknown functions (grouped together in the last line) ranged from −9.3 to −903.2.

**Table 2:** Transcripts altered in a Δ*lpp* mutant of *Y. pestis* CO92 upon temperature shift from 26°C to 37°C, compared to the wild-type strain, based on microarray analyses.

Yer ID	Gene Name	Gene Symbol	Function	Δ*lpp*/WT
				FC

*Cell envelope*				

YPO2394	major outer membrane lipoprotein	*mlpA*	*lpp* gene - cell envelope	−1138
YPO1527	putative membrane protein	—	Cell envelope	−1.7
YPO2417	putative membrane protein	—	Cell envelope	2.0

*Metabolism*				

YPO2404	conserved hypothetical protein	—	Iron-sulfur cluster assembly scaffold protein	−2.2
YPO0408	putative aldolase	—	Energy metabolism	−115.9
y1235	putative ATP-binding protein of ABC transport system	—	Inorganic ion transport and metabolism	−1.7
y0015	malate synthase A	*aceB*	TCA cycle	−2.1
y0176	succinate-semialdehyde dehydrogenase	*gabD*	Gamma-aminobutyrate metabolism	1.5

*Protein secretion and trafficking*				

YPO0502	similar to hemolysin-coregulated protein (Hcp)	—	Possible type VI secretion system effector	−52.8
YPO3275	Clp ATPase	*htpM*	Type VI secretion system clpB chaperone	−2.0
YPCD1.62	putative type III secretion regulatory protein	*lcrQ/yscM*	Blocks yop transcription	−5.3
YPO2597	sec-independent protein translocase protein	*tatE*	Protein and peptide secretion and trafficking	−1.7

*Stressor response*				

YPO3643	major cold shock protein Cspa2	*cspa2*	Stress response	3.0
y0224	cold shock-like protein	*cspI*	Stress response	3.8
y0137	serine endoprotease	*degQ*	Protease/chaperon activated in response to stress	−1.9
YPO4085	heat shock protein	*htpN/hslT*	Protein folding and stabilization, stress response	−3.5
YPO3382	global stress requirement protein GsrA	*htrA/degP*	Protease/chaperon activated in response to stress	−2.9
YPO2897	DNA-binding transcriptional regulator IscR	*iscR*	Iron-sulfur cluster assembly, stress response	−2.0
YPO0238	mechanosensitive ion channel	*mscL*	Turgor regulator, activated in response to stress	−2.5
YPO2896	putative aminotransferase	*nifS/iscS*	Iron-sulfur cluster assembly, stress response	−1.9
YPO3969	universal stress protein B	*uspB*	Stress response	−1.9

*Toxin production and resistance*				

YPO0337	similar to subtilase cytotoxin, subunit B	—	Putative toxin and probable virulence factor	−2.0
YPO0431	osmotically inducible protein Y	*b4376*	Toxin production and resistance	−55.7
YPO2333	quaternary ammonium compound-resistance protein	*qacE*	Toxin production and resistance	−4.1

*Virulence factors*				

YPO2145	similar to the *Bacillus subtilis* stage V sporulation protein R	—	Involved in spore cortex formation in *B. subtilis *	−3.2
YPPCP1.05c	pesticin	*pst*	Bacteriocin that induces the formation of spheroplasts	−1.7
YPO3138	modulating protein YmoA (histone-like protein)	*ymoA*	Protein modification and repair, invasion	−2.2

*Unknown functions*				

various genes (y1333, 1850, 3268, YPO0102, 2307, 3137, 3518, 3707, and 4064)				*

Functions were obtained from the CMR online database (http://cmr.jcvi.org) and from the literature. FC = fold-change, which was calculated as the ratio between the hybridization signal for that gene in the *Y. pestis Δlpp* mutant and WT bacteria (*Δlpp*/WT). Expression differences were deemed as statistically significant if the fold-change was ≥1.5 and *P* value ≤.05. A negative sign (“−”) before the FC indicates down-regulation in the *Y. pestis Δlpp* mutant, relative to the WT strain. *FCs for genes with unknown functions (grouped together in the last line) ranged from −1.8 to −140.6.
